# Pattern of frustration formation in the functional brain network

**DOI:** 10.1162/netn_a_00268

**Published:** 2022-10-01

**Authors:** Majid Saberi, Reza Khosrowabadi, Ali Khatibi, Bratislav Misic, Gholamreza Jafari

**Affiliations:** Institute for Cognitive and Brain Sciences, Shahid Beheshti University, G.C. Tehran, Iran; Centre of Precision Rehabilitation for Spinal Pain (CPR Spine), School of Sport, Exercise and Rehabilitation Sciences, University of Birmingham, Birmingham, UK; McConnell Brain Imaging Centre, Montréal Neurological Institute, McGill University, Montréal, QC, Canada; Physics Department, Shahid Beheshti University, Tehran, Iran; Institute of Information Technology and Data Science, Irkutsk National Research Technical University, Irkutsk, Russia

**Keywords:** Brain network, Functional brain network, Functional connectivity, Negative link, Frustration, Frustrated system, Signed network, Life-span, Subcortical regions

## Abstract

The brain is a frustrated system that contains conflictual link arrangements named frustration. The frustration as a source of disorder prevents the system from settling into low-energy states and provides flexibility for brain network organization. In this research, we tried to identify the pattern of frustration formation in the brain at the levels of region, connection, canonical network, and hemisphere. We found that frustration formation has no uniform pattern. Some subcortical elements have an active role in frustration formation, despite low contributions from many cortical elements. Frustrating connections are mostly between-network connections, and triadic frustrations are mainly formed between three regions from three distinct canonical networks. We did not find any significant differences between brain hemispheres or any robust differences between the frustration formation patterns of various life-span stages. Our results may be interesting for those who study the organization of brain links and promising for those who want to manipulate brain networks.

## Author Summary

Brain network analysis approaches commonly ignore the signs of links. Frustration is a fascinating phenomenon referring to the conflictual arrangements of signed links. As a source of instability, it can give valuable information on altering components of a network. It can specify which brain network elements intend to drive brain network alterations. Accordingly, we tried to identify the pattern of frustration formation in the brain network, which brain network elements are more frustrated and which ones are less frustrated. We provided some maps for frustration formation in the levels of region, connection, canonical network, and hemisphere. The introduced concept of frustration and our results may be interesting for brain network scientists.

## INTRODUCTION

The brain is an integrative system in that its components cooperate to execute advanced functions. So considering components of the brain independently risks oversimplification and may result in misinterpreting their role during such functions. In recent years, network modeling has been facilitating the study of the collective behavior of brain elements. Several classes of networks including simple connected-disconnected, weighted, and directed networks are being applied to clear brain mechanisms such as functional segregation and neural integration and help to discriminate brain disorders (Bassett & Bullmore, 2009; Bassett & Sporns, 2017; Liu et al., 2017a; Mišić & Sporns, 2016; Rubinov & Sporns, 2010). Most of these works ignore the sign of links, either by taking the absolute value of connections or by positive thresholding on connection values (Garrison et al., 2015; Theis et al., 2021; Wang et al., 2021). They minimize the impact of negative connections and disregard interactions between positive and negative connections.

**Figure 1.**
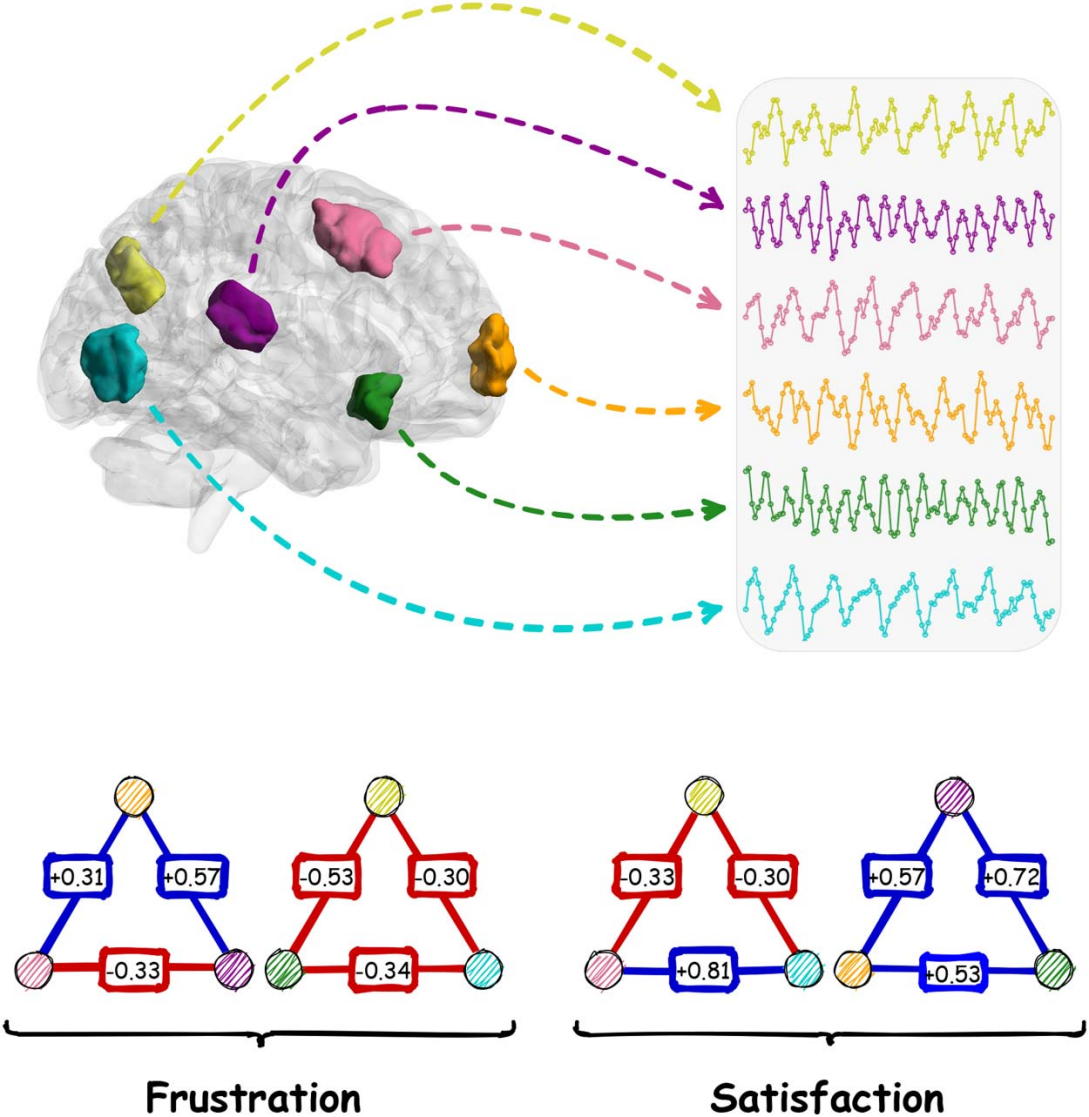
Formation of frustrated (imbalanced) and satisfied (balanced) triadic relations in the brain. Every region has a specific time course. Nodes of triads correspond to the regions by color. Correlation coefficients between every pair of activity patterns are also denoted on links. Blue and red links denote synchrony and antisynchrony between time courses.

Considering the brain as a signed network is also a promising approach to investigating the collective behavior of brain regions, where synchronous and antisynchronous coactivations of brain regions determine positive and negative links, respectively (Saberi et al., 2021a) (Figure 1). Signed networks are commonly used for social system modeling in the context of friendship and hostility between entities (Facchetti et al., 2011; Tang et al., 2016; Tomasso et al., 2022; Yang et al., 2007, 2012). Approaches such as the structural balance theory (consisted of social balance and social imbalance) are utilized to investigate signed networks for the optimized arrangement of relationships (Alabandi et al., 2021; Anchuri & Magdon-Ismail, 2012; Antal et al., 2005; Bagherikalhor et al., 2021; Derr et al., 2018; Facchetti et al., 2011). In this context, the optimized state happens when signed links have fewer higher order conflictual relations and the network has less tendency to alteration and lower energy. In this way, we recently employed the theory to study the structural balance of the resting-state network (Saberi et al., 2021a). We found that minor negative brain connections get together in a way to make negative hubs to reduce the number of conflictual signed links arrangements, which decreases brain energy and brings more stability to the brain network. Our results highlight the role of negative connections in brain network organization.

Frustration is another interesting phenomenon that can be studied in signed networks. The concept of frustration originates from the study of order-disorder systems in many-particle physics (Toulouse, 1987; Vannimenus & Toulouse, 1977; Villain et al., 1980) where it helps to understand the mechanism behind system ordering and phase transition (Goremychkin et al., 2008; Zhao et al., 2019). In spin systems, frustration is defined as topological constraints between spin neighbors that prevent minimizing system energy (Vannimenus & Toulouse, 1977). Generally, frustration as a source of disorder matters for systemic organization, alteration, and optimization.

In signed networks, frustration refers to nontrivial cycles of signed links, unstable assemblies that are seldomly found in real networks (Antal et al., 2005; Kirkley et al., 2019; Saberi et al., 2021a). Triadic relation is the smallest cycle where other elements influence the quality of a link, which makes a sense of the system (Winkler & Reichardt, 2013). It is analogous to the imbalanced triads of Heider’s balance theory (Heider, 1946; Rapoport, 1963): “the friend of a friend is an enemy” and “the enemy of an enemy is an enemy.” The theory states that entities of nontrivial relationships are frustrated about their conditions and endure pressure to change the type of relationships to become balanced: “the friend of a friend is a friend” and “the enemy of an enemy is a friend.” These conflictual arrangements have been extended to any cycles with odd numbers of negative links (Aref & Wilson, 2019; Cartwright & Harary, 1956; Estrada, 2019).

Previous works showed that the resting-state brain signed network locates in a glassy state containing triadic frustrations (Saberi et al., 2021a, 2021b). These frustrations prevent the brain network from reaching minimum energy (absolute stable state). In other words, the resting-state network is a state that can potentially transition to other brain network states. This result highlights the role of brain network frustrations in the systemic reorganization of brain links. Identifying brain network frustrations provides us with the opportunity to control the organization of brain links that affect the functionality and efficiency of the brain system. It is helpful for those who are interested in inducing functional changes by the use of neurostimulation and may promote understanding of the mechanism of brain system functions and dysfunctions. So we decided to identify the pattern of frustration formation in the brain network system in the current study. In this regard, we investigated the contribution level of brain elements including regions, functional connections, canonical networks, and hemispheres in frustration formation. We explored them for each life-span stage separately and then compared the patterns of stages.

## RESULTS

**Figure 2.**
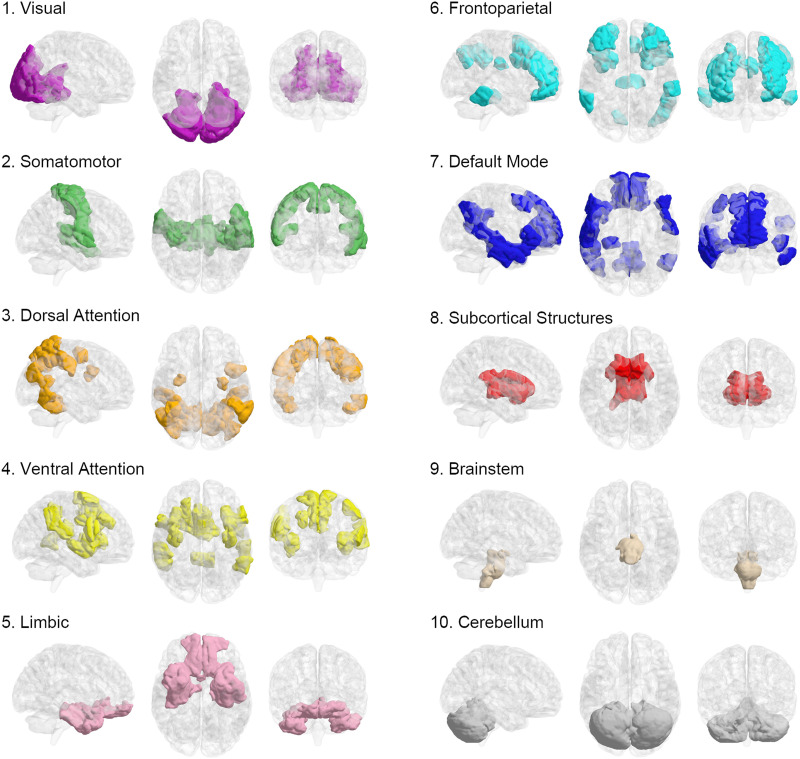
Categorizing Shen’s regions of interest into canonical networks.

**Figure 3.**
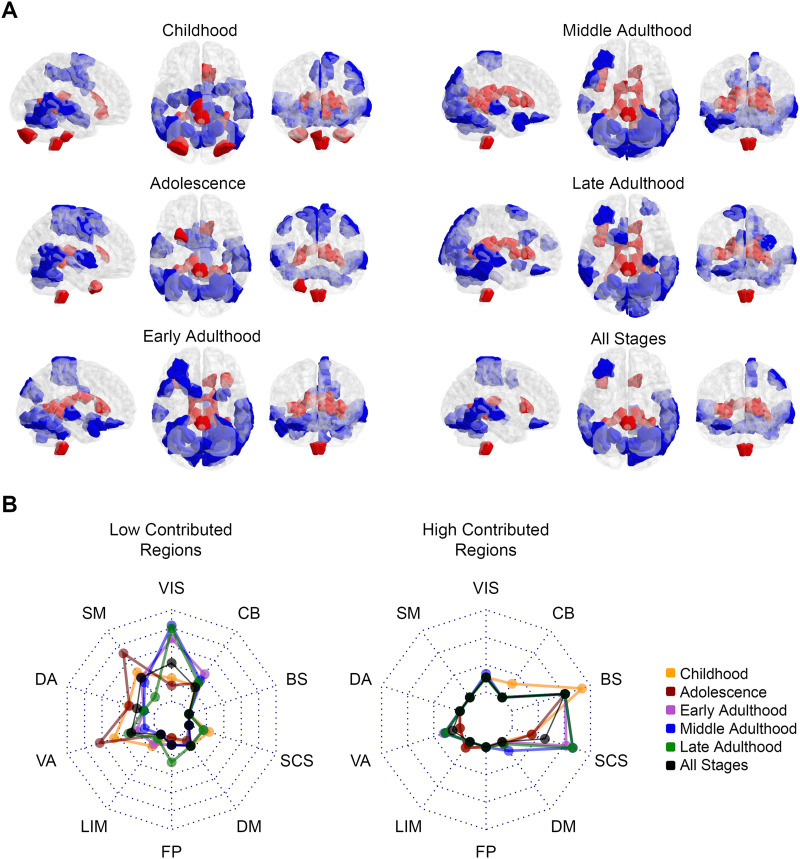
Pattern of regional contribution in frustration formation. (A) Red-colored and blue-colored areas of the brain maps indicate Shen’s regions with significantly greater and significantly lower contributions in frustration formation. Brain maps demonstrate the patterns for various life-span stages and all stages in three representational planes of sagittal, axial, and coronal. (B) Radar charts show what percentage of canonical brain networks are involved in significant areas of panel A. The left radar plot relates to blue areas and the right one corresponds to red areas. Each dashed polygon indicates 10%. Various line colors are related to life-span stages. VIS = visual; SM = somatomotor; DA = dorsal attention; VA = ventral attention; LIM = limbic; FP = fronto-parietal; DM = default mode; SCS = subcortical structures; BS = brainstem; CB = cerebellum.

**Figure 4.**
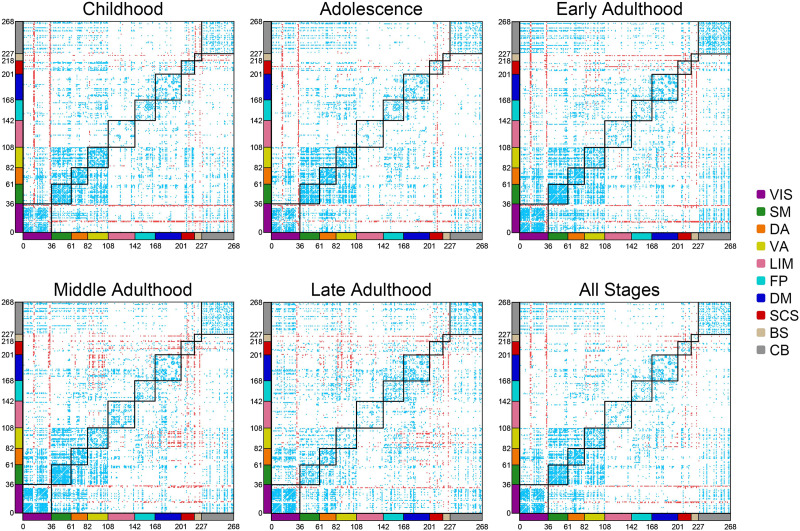
Contribution maps of functional connections in frustration formation. The first five maps demonstrate the maps related to life-span stages and the last one corresponds without considering the stage. Shen’s 268 ROIs are categorized into the 10 networks of Figure 2 by axes coloring. Cells also represent functional connections between every two ROI. Red cells display those connections with a significantly greater contribution to frustration formation, and blue cells indicate those connections with significantly lower involvement. Black squares discriminate within-network connections. VIS = visual; SM = somatomotor; DA = dorsal attention; VA = ventral attention; LIM = limbic; FP = fronto-parietal; DM = default mode; SCS = subcortical structures; BS = brainstem; CB = cerebellum.

**Figure 5.**
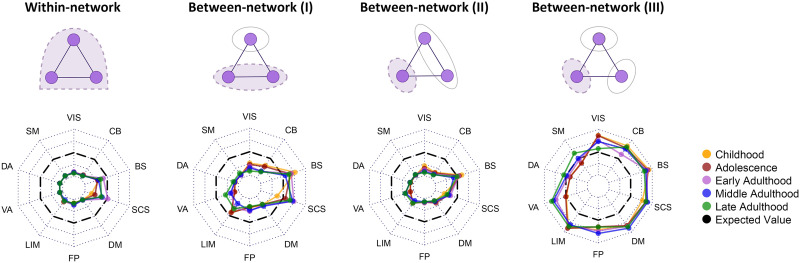
Contribution of canonical networks in frustration formation. The above row displays four possible frustration formation types between regions of canonical networks, from left to right: all regions located in the network, two regions in the network and another outside, one region in the network and two other regions in another network, one region in the network and other regions in two distinct networks. Dashed violet binds denote the elements of interest in analysis. Radar charts correspond to every frustration formation type displayed above them and show the difference between actual and expected contribution values. Dotted lines of radar charts segment *r* effect size from −1 to 1, where black dashed lines indicate zero *r* effect size. Line colors demonstrate different life-span stages. Effect size—small: |*r*| < 0.4; medium: 0.4 < |*r*| < 0.6; large: |*r*| > 0.6. VIS = visual; SM = somatomotor; DA = dorsal attention; VA = ventral attention; LIM = limbic; FP = fronto-parietal; DM = default mode; SCS = subcortical structures; BS = brain stem; CB = cerebellum.

**Figure 6.**
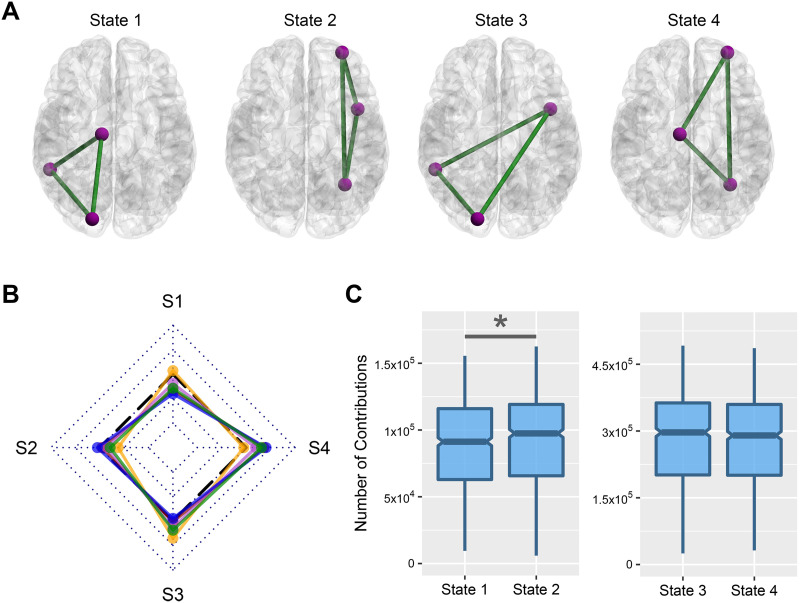
Contribution of brain hemispheres in frustration formation. (A) Four possible frustration formation types between brain hemispheres. (B) The difference between actual and null contribution values of every stage and state. Dotted rhombuses segment *r* effect sizes from −1 to 1, where black dashed lines denote zero value. S1–S4 corresponds to State 1–State 4. Lines colors—orange: childhood; red: adolescence; violet: early adulthood; blue: middle adulthood; green: late adulthood. Effect size—small: |*r*| < 0.4; medium: 0.4 < |*r*| < 0.6; large: |*r*| > 0.6. (C) Comparison between the contribution of hemispheres without considering the stage. Horizontal lines of boxes indicate medians, and notches determine the 95% confidence interval for the medians. Asterisk (*) denotes the significant corrected *p* value with a small effect size.

We designed this study to investigate the pattern of frustration formation in the brain. So we explored how brain components contribute to brain network frustrations. We preprocessed anatomical and resting-state fMRI images of healthy subjects from two online repositories, ABIDE (Di Martino et al., 2014) and Southwest University (Wei et al., 2018), which can ensure the reliability of the results. Then we extracted regional activations of each functional image based on the parcels of Shen’s atlas (Shen et al., 2013) where the regions were projected to canonical brain networks (Yeo et al., 2011) (Figure 2). After that, we calculated functional connectivities and formed a signed network for each subject based on the signs of connections. Then we identified the triadic frustrations of each network and measured the contribution level of brain regions, functional connections, canonical networks, and hemispheres in their formations. On another side, we estimated the null contribution values of mentioned elements according to the number of appeared frustrations for each subject’s signed network. Finally, we performed a group-level paired comparison for the contribution of every mentioned element between actual and null values to find out which regions, connections, canonical networks, and hemispheres were significantly involved in the frustration formation for each stage separately (Figure 3–6). Finally, we provided a multimodal map for frustration formation in the brain stagewise and without considering stages and investigated any significant differences between contribution patterns of life-span stages.

### Contribution of Brain Regions

For each subject, we identified presented frustrations and determined the number of frustrations that each region is involved in. We also calculated the expected values of regional involvements in the case of a uniform engagement of regions in frustration formation. The expected contribution value of a region of interest (ROI) for a subject’s whole-brain network with *N*_*ROI*_ regions and *N*_*Frust*_ frustrations is derived as follows:

$$\frac{3}{N_{ROI}}\times N_{\text{Frus}}$$

Number 3 is multiplied since a frustration occurs between three regions. In this way, we provided actual and null values of contributions in frustration formation for 268 regions of each subject. Then we performed a paired group-level comparison between actual and null values of each region to find which regions have a significantly different contribution. We analyzed subjects of each stage separately in addition to disregarding the life-span stages (Figure 3). Since contribution values were distributed nonnormally, we used Wilcoxon matched-pairs signed-rank test for this goal. Figure 3A indicates regions with significantly lower (blue-colored) and significantly greater (red-colored) involvements in frustration formation compared to expected values after multiple comparison corrections on *p* values using false discovery rate (FDR) and with large effect sizes (greater than 0.6). We reported the corrected *p* values and effect sizes in the Supporting Information. Since the frustration expresses disorganization in the network, the blue and red areas of the figure represent regions that have well-organized and disorganized relations with other regions, respectively. To better interpret the results, we mapped significant brain regions to canonical networks of Figure 2 to show how red and blue areas belong to the canonical networks. The Methods section “Parcellation Atlas Projection” describes the projection process that we used to obtain Figure 2 in detail. Radar plots of Figure 3B represent what percentages of canonical networks have significant contributions where dotted polygons divide the whole network volume by 10%. The left and right radar charts correspond to blue-colored and red-colored regions of Figure 3A. The left radar chart shows low contributed blue regions mostly belong to the visual network in adulthood and they involve somatomotor and ventral attention networks in the early stages. More than 40% of the visual network in middle adulthood and about 35% of the somatomotor network and ventral attention network in adolescence have regions with well-organized relations to other brain regions. The right radar chart also shows that most of the high contributed red regions of Figure 3A belong to subcortical structures and the brain stem. Despite early stages, more than 20% of subcortical structures in adulthood have areas with disorganized relations to other regions. Also, more than 20% of the brain stem volume is significantly involved in frustration formations, an inferior portion of the brain stem according to Figure 3A. For all stages on the brain map, radar charts indicate moderate values, which we desired in case of disregarding stages. We also used the nonparametric Kruskal–Wallis test to investigate between-stage differences of regional contributions. We performed the test between contribution values of various stages for each region separately and then corrected *p* values from false positives that occurred by multiple comparisons using FDR besides effect size measurement. There is no region with a significant corrected *p* value and large effect size (greater than 0.14) that indicates no robust contribution differences between stages. Although, Supporting Information Figure S1 shows regions with corrected *p* value lower than 0.05 and medium effect size (between 0.06 and 0.14). To test whether significant regions of Figure 3A appears randomly or not, we compared the entropy of the all stage pattern with the entropy of its shuffled pattern for greater and lower contributions separately. We explained the way we calculated entropies in the Methods section “Entropy Calculation.” Supporting Information Figure S2 shows the histogram of entropies related to shuffled patterns compared to the entropy of actual patterns. It indicates that both lower contributed regions (blue-colored) and greater contributed regions (red-colored) have significantly lower entropies that validate the nonrandomness of the regional significant patterns of Figure 3.

### Contribution of Functional Connections

After regional investigations, we explored the contribution of functional connections in frustration formation. So for each subject, we counted the number of triadic frustrations that each connection is involved in. Also, we estimated the expected contribution values of each connection in case of uniform involvement of connections as follows:

32NROI×NFrus

where *N*_*Frust*_ and *N*_*ROI*_ are the number of presented frustration and the number of nodes in the subject’s network. 2NROI also denotes the 2-combinations of *N*_*ROI*_ that is equals to the number of links in a fully connected network with *N*_*ROI*_ regions. The factor of 3 also appears because frustration engages three connections. In this way, we calculated actual and null contribution values for each connection of each subject. After that, we performed a paired group-level analysis between actual and null values for each functional connection using Wilcoxon matched-pairs signed-rank test. We chose this nonparametric test since the values were not distributed normally. Figure 4 displays contribution maps of functional connections in frustration formation for each stage separately and for all stages. Each cell corresponds to a functional connection between two regions of interest, so heat maps represent mutual connections between 268 of Shen’s regions. The regions are categorized based on networks of Figure 2 by segmenting the axes of heat maps. Also, black squares discriminate between-network connections. Blue and red cells indicate connections with significantly lower and significantly greater contributions in frustration formation that have FDR corrected *p* values smaller than 0.05 and large effect sizes (greater than 0.6). The figure does not exhibit a distributed pattern of high contributed connections (red-colored cells). It seems that connections of several visual and subcortical regions with other regions have significantly great contributions to frustration formation. Many default mode to ventral attention connections of adulthood are also high contributed. On the contrary, lower contributed connections are more distributed in the brain network. Most of these connections are within-network type, especially those located in visual, somatomotor, and attentional networks. There are also lots of between-network connections that have lower contributions in frustration formation, especially connections between visual, somatomotor, cerebellar, and attentional regions. We reported corrected *p* values and effect sizes of the comparisons in the Supporting Information.

As well as the last section, we also wanted to explore the randomness of the pattern of the significantly contributed connections. Based on what we described in the Methods section, we estimated the entropy of the all stage subfigure of Figure 4 and its shuffled patterns for significantly lower connections (blue-colored) and significantly greater connections (red-colored) separately. Supporting Information Figure S3 shows that the pattern of blue-colored connections and the pattern of red-colored connections have lower entropies compared to shuffled patterns that states significant connections are not random.

In addition, a question has arisen whether high contributed and low contributed connections that are denoted in Figure 4 are within-network type or between-network type. To answer this question and better interpret the heat maps of Figure 4, we created a metric and named it WBR. WBR measures the ratio of within-network connectivity to between-network connectivity. As such, we first consider our interesting connections, for example, higher contributed connections, then count the number of appearing between-network connections and within-network connections. On another side, we calculate the number of possible between-network connections and within-network connections based on the number of nodes. Finally, we divide the calculated values as below:

$$WBR=\frac{\frac{WC}{BC}}{\frac{{WC}_{0}}{{BC}_{0}}}$$

where *WC* and *BC* stand for the number of within-network connections and the number of between-network connections, and *WC*_0_ and *BC*_0_ are also their maximum possible values. *WBR* would be more than one in the case of intense within-network connections, lower than one in the case of extreme between-network connections, and equal to one in the balance between them. Table 1 demonstrates *WBR* of high contributed connections and low contributed connections in frustration formation calculated based on heat maps of Figure 4. The results show that the low contributed connections tend to be within-network type and high contributed connections are mostly between-network type. We also investigated the difference in the contribution of connections in frustration formation between life-span stages. So we performed a multigroup Kruskal–Wallis test for each connection between observed contribution values of different stages subjects. Then we corrected multiple comparison effects on *p* values using the FDR method. We did not find any significant corrected *p* values with large effect sizes (greater than 0.14) for various functional connections. Supporting Information Figure S4 only shows connections with corrected *p* values lower than 0.05 with medium effect sizes (between 0.06 and 0.14).

**Table 1.** WBRs calculated based on low contributed and high contributed connections of Figure 4 heat maps

Functional connections	Childhood	Adolescence	Early adulthood	Middle adulthood	Late adulthood	All stages
Low contributed (blue cells)	3.962	4.239	4.163	4.579	3.903	4.966
High contributed (red cells)	0.507	0.718	0.615	0.273	0.253	0.269

### Contribution of Canonical Networks

In the last sections, we explored the involvement of brain regions and their functional connections in frustration formation. In this section, we wanted to study the contribution of canonical networks. So we used the projection of Shen’s ROIs into Yeo’s networks (Figure 2 and Methods section “Parcellation Atlas Projection”). There are four types of frustration formations that regions of a canonical network can be involved in (Figure 5), (1) within-network: all three regions belong to the target network; (2) between-network (I): two regions located in the target network and one another outside; (3) between-network (II): one region located in the target network and two other regions in another network; and (4) between-network (III): one region located in the target network and two other regions in two other distinct networks. For every combination of formation type, stage, and canonical network, we performed a pairwise group comparison using the Wilcoxon matched-pairs signed-rank test between actual and expected contribution values of subjects and then integrated all results into Figure 5 and Supporting Information Tables S1–S4. Formulas to calculate null contribution values of the target network of *T* in mentioned frustration formation types are described as follows:

3NT3N×NFrus(within-network)

where *N* and *N*_*T*_ denote total ROI number and those ROI numbers located in canonical network of *T*; 3N and 3NT equal to the possible triangle numbers in whole-brain network and the possible triangle numbers in the target network. *N*_*Frus*_ is also the number of appeared frustrations in corresponding actual whole-brain network and we wanted to calculate its null values.

2NT×N−NT3N×NFrus(between-network(I))

The numerator of the fraction is equaled to the number of triangles that their two ROIs located in the target network of *T* and one another outside.

∑i≠TNT×2Ni3N×NFrus(between-network(II))

The summation performs over all the networks except network of *T*, and the numerator of the fraction is equaled to the number of triangles that their one ROI located in the target network and two ROIs in another networks.

∑i≠j≠TNT×Ni×Nj3N×NFrus(between-network(III))

The summation performs over every distinct pair of networks except network of *T*. The numerator of fraction denotes possible triangles that one of their ROIs is located in the target network and two other ROIs belong to two other distinct networks. After counting contribution values for formation types and calculating null values, we performed paired group analysis. All the effect sizes and corrected *p* values are reported in the Supporting Information. Radar charts of Figure 5 demonstrate the *r* effect size of Wilcoxon matched-pairs signed-rank tests between actual and null contribution values. The results demonstrate that all networks in all of the stages except all stages’ brain stem and adults’ subcortical structures have significantly lower within-network types of frustration formation with large effect sizes (greater than 0.6). Visual, somatomotor, dorsal, and ventral attention, fronto-parietal, and default mode networks have significantly lower contributions in between-network frustration formation type I, although their effect sizes are medium (between 0.4 and 0.6) in some stages. Also, early adults’ limbic networks and adults’ cerebellums have significantly lower contributions in between-network type I and large effect sizes. Subcortical structures in early and middle adulthood and brain stem in childhood have significantly higher contributions in the formation of between-network type I with medium effect sizes. Investigation of between-network type II also shows that most of the networks in all stages have significantly lower contributions in frustration formation with large effect sizes except brain stem and subcortical structures, where subcortical structures just in childhood, adolescence, and late adulthood have medium effect sizes. The last radar chart (between-network III) shows that most of the higher contributions occur between three distinct networks. Although, it indicates that somatomotor and dorsal attention are not contributed to this type of frustration formation. Most other networks of all stages have significantly higher contributions in this type of frustration formation with large or medium effect sizes except visual network in late adulthood, ventral attention in childhood and adolescence, and fronto-parietal in late adulthood that have no significant *p* values, and fronto-parietal in adolescence and cerebellum in early adulthood that have small effect sizes. We also compared contribution values of different stages for every pair of formation types and canonical networks using the Kruskal–Wallis test. The test obtained no significant corrected *p* values with large effect sizes. *P* values and effect sizes of these analyses are also presented in the Supporting Information.

### Contribution of Hemispheres

In the end, we investigated the contribution of the brain hemispheres in frustration formation. There are four possible states for the contribution (Figure 6A): (1) all three regions of frustration belong to the right hemisphere, (2) all three regions belong to the left hemisphere, (3) only two regions located in the right hemisphere, and (4) and only two regions located in the left hemisphere. We compared actual and null contribution values for every combination of state and stage using the Wilcoxon matched-pairs signed-rank test. We estimated the expected contribution values of four states for a network as follows:

3NLH3N×NFrusState1

3NRH3N×NFrusState2

2NLH×N−NLH3N×NFrusState3

2NRH×N−NRH3N×NFrusState4

where *N*_*RH*_ and *N*_*LH*_ are the number of regions in right and left hemispheres that are equaled to 133 and 135 according to Shen’s atlas. Figure 6B shows the result of comparisons. We reported their statistics in Supporting Information Table S5. The results show no significant corrected *p* values with large effect size (greater than 0.6) for the comparisons. All effect sizes were small except State 4 in middle adulthood that has medium effect sizes (*r* effect size = 0.408). We also compared contribution values of different stages in every state and did not find any nonsmall effect sizes for between-stage differences. To investigate the main effect of the hemisphere on frustration formation disregarding life-span stage, we also performed two Wilcoxon matched-pairs signed-rank tests, one between State 1 and State 2 contribution values and the other between State 3 and State 4 contribution values (Figure 6C). Only the comparison between State 1 and State 2 had a significant *p* value, although its effect size was small (*r* effect size = −0.38). Generally, we did not find any robust hemispherical effect on frustration formation.

## DISCUSSION

We wanted to find out the role of different brain elements in frustration formation. We compared the number of frustrations that each element contributes with the null contribution value of that element. The null values are estimated based on the hypothesis of the uniform contribution of the elements in frustration formation. We performed the comparison in four levels of the brain region, functional connection, canonical network, and hemisphere. We did it for each life-span stage separately as well as without considering stages. We also compared the involvement of different stages. Regional level comparisons show that some brain regions have greater contributions, and some regions have lower contributions in frustration formation; although, we did not find any robust differences between the regional contributions of different stages (Figure 3). Investigation of functional connections also indicated that many functional connections have significantly lower or greater contributions to frustration formation (Figure 4). Low contributed connections are mostly within-network types and high contributed connections are usually the between-network type (Table 1). We also did not find any robust variation in the contribution of connections between stages. In addition, the results show that the most significant regions and connections are lower contributed. Entropy analysis also indicated that regional and connectional significant patterns are not random. In the following, we studied the contribution of canonical networks in frustration formations. We found that most frustrations appear between three regions of three distinct networks (Figure 5), and there are no powerful differences between-network-based contributions of life-span stages. We also did not find any strong hemisphere-related effects on frustration formation and no difference between the role of the right and left hemispheres (Figure 6).

### Role of Subcortical Structures

Our results suggest that subcortical regions have a prominent role in frustration formation in the brain network at both nodal and connectional levels. So they can bring instability and altering properties that facilitate systemic level neural changes and provide adaptive characteristics to the brain. Certainly, we cannot understand brain reorganization without taking into account the white matter tract located in subcortical areas. Older theories considered subcortical alterations as a passive consequence of cortical functional reshaping; however, new mechanisms underline the active reorganization of subcircuits within the large network of the brain (Duffau, 2009). Animal studies posited brain stem–related and subcortical plasticity under exposure to visual and auditory stimuli (Chandrasekaran et al., 2014; Duménieu et al., 2021; Miranda et al., 2014). Human studies reported such subcortical rewiring mechanisms for skill learning such as second language and motor training (Liu et al., 2020; Sampaio-Baptista et al., 2013; Scholz et al., 2009). A recent study also indicates subcortical short-term plasticity yielded by deep brain stimulation pulse (Awad et al., 2021). All these observations are agreeing with the frustrating essence of the brain subcortex. In addition, we know that many studies reveal malfunction and abnormal structural changes in the subcortex of neural disorders (Arnold Anteraper et al., 2014; Cerliani et al., 2015; Hoogman et al., 2017; Lee et al., 2018; Rosenberg-Katz et al., 2016) while subcortical small changes may have extensive effects at the cortical level (Jones, 2000). Therefore, controlling the activation of frustrating subcortical regions can provide a favorable systemic reorganization of the brain network. It may be appliable by focusing on our detected frustrating regions and connections using sophisticated subcortical stimulation methods (Awad et al., 2021; Colle et al., 2021; Folloni et al., 2019; Shi et al., 2021). In addition, the prominent role of the subcortex in frustration formation may be related to its unidirectional projections (Shi et al., 2014) or indirect pathways (Lanciego et al., 2012), which need more exploration.

### Between-Network Frustrations

The brain is a self-organized system (Dresp-Langley, 2020) such that functional signed links adopt a topology to reduce network frustration (Saberi et al., 2021a). We found that this property is consistent within canonical networks as well, where canonical networks have lower numbers of frustrations compared to null (Figure 5). Most of the frustrating connections are formed between networks (Table 1), and frustrated triangles mostly engage three distinct networks (Figure 5). This is what we expect to happen since large-scale brain networks consist of coactivating local brain seeds (Yeo et al., 2011). They decrease the chance of negative functional connection appearance and subsequently frustration formation. Consequently, if someone is interested in handling brain network frustration he/she should pursue a multinetwork approach.

### Visual Network Contribution

Our results indicate that most visual regions of adults have a low contribution to frustration formation (Figure 3), which may be a consequence of visual maturation (Siu & Murphy, 2018). Although, several ROIs belonging to the hippocampus show frustrating connections to most brain areas (Figure 4). It is an interesting result since the prominent role of the hippocampus in learning and memory and its association with broad areas of the brain to play this role (Anand & Dhikav, 2012) are consistent with the frustrating essence of its connections. We should mention that frustration facilitates adaptive processes due to their unstable nature.

### Somatomotor Network Contribution

As Figure 3 shows, nearly 40% of somatomotor and ventral attention regions of adolescents have a lower contribution to frustration formation than adults. We wonder why this happens while neural flexibility is an essential developmental feature of adolescence. We could not find a proper answer or any biological relevance for this effect.

### Ventral Attention to Default Mode Connections

We also saw some ventral attention to default mode connections are frustrating (Figure 4). This effect is enhanced in the adulthood range. We could not find a good psych-neural interpretation for this observation. Although, a recent paper highlighted connectivity between ventral attention and default mode areas in Bulimia Nervosa (Domakonda et al., 2019). Because of the high comorbidity of Bulimia Nervosa with other mental disorders such as depression and bipolar (McElroy et al., 2005; Walsh et al., 1985) and the frequency of mood disorders in adulthood range, we think the frustrating properties of these connections may be related to these types of disorders.

### Life-Span Comparisons

In one of our previous works, when we compared the number of frustrations between life-span stages to track the requirement to change of brain network, we reported a significant difference between stages (Saberi et al., 2021b). Because of the large sample size of stages, we decided to consider effect size besides corrected *p* value in current work comparisons to specify more robust differences. We did not find any strong differences between frustration formation patterns of various stages; in other words, all significant comparisons with corrected *p* values lower than 0.05 had corresponding small and medium effect sizes.

### Shen’s Atlas to Yeo’s Atlas Projection

As we wanted to investigate the quality of canonical brain networks’ contribution to frustration formation, we needed projection of Shen’s 268 ROIs into them. Finn et al. (2015) utilized Shen’s clustering algorithm (Shen et al., 2013) to group the ROIs into eight networks of the medial frontal network, fronto-parietal network, default mode network, subcortical and cerebellar regions, motor network, visual I network, visual II network, and the visual association network. The labeling is presented at https://bioimagesuiteweb.github.io/webapp/connviewer.html and has been used for the network-based analysis of studies (Chen et al., 2020; Rosenberg et al., 2016). When we explore the output of the categorization, we can observe many implausible labeling, for instance: some superior regions labeled as subcortical and cerebellar networks, some temporal regions categorized into medial frontal, and some cerebellar regions classified as fronto-parietal, visual, and default mode networks. Also, the limbic network, which is one of the most important functional brain subsystems, is not regarded. Since three networks are nominated based on the sense of vision, a question has arisen: if the sensory system has been considered a major element of the labeling, why is there no sign of other sensations such as auditory in the ROIs categorization. As there seem to be some shortages in the categorization process, we decided to use a new standard procedure (Lawrence et al., 2021) to project Shen’s ROIs into Yeo’s seven large-scale brain networks (Yeo et al., 2011) based on the coincidence index of the two atlases (Supporting Information Figure S5). Since Yeo et al. (2011) excluded subcortical areas, we specified them into three distinct networks according to their anatomical characteristics. We described the procedure and the intermediate results in the Methods section “Parcellation Atlas Projection.” So our optimized model classified Shen’s regions into 10 subnetworks of visual, somatomotor, dorsal attention, ventral attention, limbic, fronto-parietal, default mode, subcortical structures, brain stem, and cerebellum (Figure 2).

### Global Signal Effects

There is much conflictual evidence on the quality of removing global brain signals; some studies suggest this step in preprocessing and some others decline it (Aquino et al., 2020; Fox et al., 2009; Liu et al., 2017b; Murphy et al., 2009; Schölvinck et al., 2010). It is clear that global signal regression produces such antisynchronous connections with negative correlations (Fox et al., 2009) (Supporting Information Figure S6) that matter for signed networks and can affect the structural balance. It is also a fact that increasing negative links grows the number of frustrations since they have more negative links as compared to satisfaction (Figure 1). In the analysis of the main manuscript as well as our previous research (Saberi et al., 2021a, 2021b), we ignored global signal regression and explored the formation of frustration in the presence of minor negative links. Besides, we performed the same analysis on the global signal regressed functional images to check the difference. Supporting Information Figures S7 and S8 show the result of the analysis for the contribution of regions and contribution of connections in frustration formation. Figure S7 shows a lower number of significant regions compared to Figure 3 and indicates some other regions. Low contributed regions belong to somatomotor and attentional networks in adulthood and default mode in all stages, and high contributed regions are mostly located in the subcortex in early adulthood and outspread in other stages. Investigation of connection contribution on the regressed images (Supporting Information Figure S8) also shows fewer numbers of significant connections compared to Figure 4, in which most of them are lower contributed and within-network type. In summary, it seems that global signal regression gives little but different information about frustration formation. In addition, we did not find any significant differences between frustration formation of life-span stages as well as without the global signal regression approach.

### Parcellation-Based Reliability

Many parcellation atlases were developed based on the anatomical and functional attributes of the brain and using different algorithms. In connectivity studies where brain parcellations are used, a question always arises about whether the results are reliable under other atlases (Domhof et al., 2021; Popovych et al., 2021). Actually, we utilized Shen’s parcellation atlas (Shen et al., 2013) and categorized its 268 regions into canonical networks (Yeo et al., 2011) in our analysis. To check the mentioned reliability, we chose Desikan–Killiany–Tourville (DKT) atlas (Klein & Tourville, 2012) as one of the most similar ones to Shen’s parcellation with 101 cortical and subcortical ROIs, although it has different ROI boundaries and lower resolution and was developed based on another algorithm. We also categorized DKT ROIs into canonical networks based on the projection manner that we explained in the Methods section and Supporting Information Figure S9. Since our analysis was focused on brain elements and considering elemental features of brain atlases are not the same in various atlases, obtaining similar global features and moderate similar local features may be satisfying. Supporting Information Figure S10 shows regional contribution analysis for DKT ROIs and Figure S11 demonstrates that for corresponding functional connections. Both of them do not indicate any significant differences between the contribution map of life-span stages, as the same results were obtained based on Shen’s atlas. Also, subcortical regions and their connections with other brain regions show high contributions to frustration formation that emphasizes the effective role of the subcortex. Most low contributed connections are also within-network types and high contributed connections are almost between subcortical structures and other brain regions. Somatomotor and visual areas also show a low contribution to frustration formation. However, we can see contradictory observations compared to Shen’s related results. For example, a medial large region of the default mode network shows a high contribution opposite to Shen’s results, and the limbic network has some low contributed regions. The differences may be routed in the dissimilarity of the atlases.

### Neural Essence of Frustration

Frustration is formed by a combination of positive and negative links (Figure 1). Positive and negative links refer to regional synchronous and regional antisynchronous coactivations, respectively. We do not know which mechanism is behind appearing negative links; maybe it is related to time delay due to axonal propagation where influential subcortical regions that are highly contributed to frustration formation have large axonal wiring (Petkoski & Jirsa, 2019; Petkoski et al., 2018). It is an open question that needs more investigation, and computational modeling can be helpful to solve it. Positive and negative links conceptually look the same as inphase and antiphase synchronizes (Petkoski & Jirsa, 2019, 2022; Petkoski et al., 2016), whereas Petkoski and his colleagues showed that inphase synchrony (or perfectly aligned in/antiphase clustering) makes the lowest energy that is similar to a brain signed network that has no negative links and frustrations located in the lowest balance energy. They also found that when giving the distribution of the time delays in the brain, it is more probable that the brain minimizes the disorders that are consistent with our previous results (Saberi et al., 2021a) and the self-organizing essence of the brain (Dresp-Langley, 2020). So we propose their computational approach as a high-potential way to investigate the mechanism behind negative link and frustration appearances.

### Frustration as a Brain Network Measure

Some graph measures are defined based on the arrangement of graph links; for example, we can calculate the clustering of a network by counting triangles, and motifs as subgraphs are considered the building blocks of the networks. They are commonly investigated in brain networks (Liu et al., 2017a; Rubinov & Sporns, 2010) and extracted from connected-disconnected graphs where either we have a link between two nodes or we do not have any. Although frustration is defined in a signed network where presented links have two states of positive and negative and gives information on the system disordering to us.

### Disregarding Threshold on Connections

Thresholding is a common way of making a brain network (Garrison et al., 2015; Theis et al., 2021; Wang et al., 2021). To provide a brain signed network, we claim that although a functional brain network has a low number of antisynchronous coactivations (Supporting Information Figure S6), the impact of positive and negative connections are not the same, so we should not consider the same threshold for both of them, so disregarding the thresholding process is a good way. Consequently, a question emerges that maybe most negative links randomly appear. To answer it we checked the randomness of the contribution pattern (Figures S2 and S3). Since we found that they are not random we can conclude that disregarding the threshold is not vulnerable and negative connections’ appearance is meaningful.

### Conclusion

In summary, many brain elements play an active role in the frustration formation of the brain network; however, many other elements are less contributed to frustration formation. We identified both of them at the level of the node, connection, and network. The subcortical areas and hippocampus are the most influential region for frustration formation. Matured visual regions have less propensity to get involved in frustrations. Ventral attention to default mode connections are frustrating, especially in adulthood. Generally, regional and connectional contribution patterns are not random, and the frustrations are mainly formed between three distinct networks. Also, there is no robust difference between the contribution pattern of brain elements in frustration formation between life-span stages. The study of network frustration can reveal the mechanisms behind neural alteration and brain disfunction. Localization of the frustrations also provides the possibility of brain network reorganization.

## METHODS

### Neuroimage Data

We collected functional and anatomical T1 images from two public repositories of ABIDE (Di Martino et al., 2014) and Southwest (Wei et al., 2018). The Southwest database contains early adulthood to late adulthood subjects, and ABIDE contains childhood to early adulthood subjects. We selected all healthy subjects whose functional repetition times were equal to 2 seconds (most frequent repetition times). Selected subjects aged from 6 to 80 (mean: 31.31; *SD*: 19.78) and 44% were female. We classified subjects into five life-span stages of childhood (age: 6–12), adolescence (age: 12–18), early adulthood (age: 18–40), middle adulthood (age: 40–65), and late adulthood (age: greater than 65) according to Erikson’s stages (Sharleen, 2013). After preprocessing, we also excluded subjects whose images could not pass the quality check. Supporting Information Table S6 represents the demography of 793 finalized subjects based on the stage and neuroimaging site. Table S7 also describes site-specific imaging protocols. We should mention that the neuroimaging procedures were carried out in compliance with the Declaration of Helsinki. All adult subjects and parents (legal guardians) of subjects under the age of 18 provided informed consent before starting the procedure. Neuroimages are collected in several sites, and the acquisition protocols were approved by their licensing committees including the Research Ethics Committee of the Brain Imaging Center of Southwest University, Institutional Review Boards of the New York University School of Medicine, The Institutional Review Board of San Diego State University, the Institutional Review Board of University of Michigan, Yale University Institutional Review Board, Ethics Commission of ETH Zurich, Georgetown University Institutional Review Board, Hospital of Trinity College, and The Institutional Review Board of University of Utah School of Medicine.

### Preprocessing Functional Images

We employed FSL (Jenkinson et al., 2012) and AFNI (Cox, 1996) to preprocess images. At first, we extracted the brain tissue from the T1 image, then segmented it into gray matter (GM), white matter (WM), and cerebral spinal fluid (CSF). Then we removed the first five volumes of the functional image to assure magnetic stability and then performed slice timing correction. After that, we registered volumes of the functional image to the extracted brain of the T1 image using the least square optimization with three translational and three rotational variables. Then we conducted spatial smoothing on registered volumes using a Gaussian kernel (FWHM = 5 mm). In the following, we interpolated spiking outliers of every voxel’s time series and applied band-pass filtering (0.01–0.09 Hz) to them to exclude nonrelevant information. We also regressed out three translational and three rotational confounds of motions as well as WM and CSF signals from the time series of every voxel. Finally, we normalized volumes of functional images to MNI152 standard space (2 × 2 × 2 mm^3^) by optimization of 12 variables including 3 translational, 3 rotational, 3 scaling, and 3 shearing variables. We did not regress out the global signal from functional image for analysis of main manuscripts, although we brought a version of the results that regressed out global signal from the image in the Supporting Information. In the end, we inspected the quality of preprocessing. So we excluded subjects whose images had low extraction and registration quality and those with movement parameters greater than one voxel size. It is beneficial to mention that we had used the procedure in other studies (Saberi et al., 2021a, 2021b; Sadeghi et al., 2017).

### Regional Brain Activations

We used MATLAB software to extract 268 regional activity patterns from every preprocessed functional image based on Shen’s atlas (Shen et al., 2013). Although all functional images were acquired with the same repetition time, imaging sites had different acquisition times, volume numbers, and regional time points. The shortest acquisition time belongs to the Georgetown University site of ABIDE with 147 volumes. Accordingly, we chose 147 first time points of all regional time series. So we obtained 268 time series with 147 time points as activity patterns for each subject. Since all repetition times were equal, adjusted activity patterns corresponded to an equal acquisition time. The equality of the number of time points and equality of acquisition times matter in connectivity matrix formation and help to improve the validity of the comparisons.

### Frustration Formation

We constructed a connectivity matrix for each subject according to Pearson’s correlation of pairs of regional time series. We only considered signs of correlation coefficients to obtain the adjacency matrix of the subject’s signed networks denoting positive and negative links. Then we identified the triadic frustrations of the subject’s networks. After identifying frustrations, we counted the number of triadic frustrations that every element contributed to its formation. We measured the contribution value for every nodal, connectional, network-based, and hemisphere element. We explained the way to estimate the expected contribution values of the elements in the Results section.

### Structural Balance Theory

Attitude change is a social psychology topic that studies how people change their beliefs about concepts and objects. Fritz Heider’s attitude change theory is known as the balance theory (Heider, 1946), a triangle between two individuals and one object. It describes how the relationship between individuals affects the quality of individual attitude toward the object that we consider trivial nowadays, for example, business owners introduce their products by using social influencers to better change the attitude of customers. Heider’s theory was extended to interpersonal relations: when a friend’s friend or an enemy’s enemy is a friend, the triadic relation is balanced; and when a friend’s friend or an enemy’s enemy is an enemy the triadic relation is imbalanced (Rapoport, 1963). The imbalanced triads are nontrivial and unstable, so their entities are frustrated about their condition and try to alter their relationships to become balanced (Heider, 1946). Generally, components of a balanced triad are satisfied with their situation, and components of an imbalanced triad are frustrated about their situation (Aref & Wilson, 2019; Estrada, 2019). Also, Cartwright and Harary (1956) generalized Heider’s theory to a wider range of interactions. They developed structural balance theory using mathematical graphs. In this context, we can explore the balance of a system containing lots of positive and negative interactions. In recent years, network scientists used the structural balance theory to analyze various signed networks with a large set of entities and signed links (Anchuri & Magdon-Ismail, 2012; Kirkley et al., 2019; Saberi et al., 2021a; Zahedian et al., 2022). In this context, we can define energy and phase states for the signed network, discriminate between different state types, and investigate criticality and transitions between states (Antal et al., 2005; Bagherikalhor et al., 2021; Derr et al., 2018; Marvel et al., 2009).

### Parcellation Atlas Projection

As we wanted to study the contribution of the canonical networks in frustration formation, we needed to know which Shen’s ROIs belong to which canonical networks. So we calculated the Dice coefficient between any pair of Yeo’s networks and Shen’s ROIs as follows (Dice, 1945; Lawrence et al., 2021):

$$C_{ij}=\frac{2h_{ij}}{a_{i}+b_{j}}$$

where *a*_*i*_ and *b*_*j*_ correspond to the total voxels in region *i* of Shen’s atlas and the total voxels in network *j* of Yeo’s atlas, respectively; *h*_*ij*_ also denotes the total number of overlapped voxels between them. So we obtained a matrix where each cell indicates a Dice coefficient for a pair of ROI and canonical network (Supporting Information Figure S5). According to the values, we estimated that every ROI belongs to what network based on the largest coefficient. Since Yeo only parcellated the cerebral cortex, we grouped subcortical ROIs into three networks of subcortical structures, brain stem, and cerebellum based on their anatomical information. Figure 2 shows the result of ROI categorization. We attached labeled ROIs in the Supporting Information. In the same way, we also categorized regions of DKT atlases (Figure S9) and provided them to check reliability of results under changing parcellation.

### Entropy Calculation

Entropy is a measure of system randomness. When a system has some possible microstates with identical probabilities, Shannon’s entropy determines randomness of variable *X* as follows:

$$H\left(X\right)=-\sum_{i=1}^{n}P\left(x_{i}\right)\;\log P\left(x_{i}\right)$$

where summation applies on all states and *P*(*x*_*i*_) denotes the probability of the state *x*_*i*_. The entropy is maximum in the case of equality of states probabilities. We wanted to know whether the appearance of significant nodes and connections in Figure 3 and Figure 4 are random or not. So we calculated Shannon’s entropy for actual patterns and compared them with the entropy of 1,000 shuffled patterns. In this regard, we measured the entropy of regional patterns as follows. Firstly, we considered canonical networks as states, counted the number of significant regions located in each network, normalized counted numbers to bin size (total number of ROIs located in networks), then divided the outputs by the total number of significant ROIs. In this way, we provided probabilities of states and calculated Shannon’s entropy for the apparent pattern of significant brain regions (Figure S2). Similarly, we measured Shannon’s entropy for the pattern of significant brain connections, but we regarded connections between networks as states (Figure S3).

### Statistical Analysis

In this study, we had two types of comparison for each element: the two-group paired comparison between actual and null contribution values, and the multiple-group comparison between contribution values of life-span stages. We used Wilcoxon matched-pairs signed-rank test for the first one and the Kruskal–Wallis test for the last one. We utilized nonparametric statistical analysis because contribution values were not distributed normally. We performed multiple comparison corrections on *p* values using FDR and considered effect sizes to improve the validity of the statistical analysis. We also used two different algorithms for calculating effect sizes of between-group and multiple-group analyses with different thresholds (Table 2). Also, we calculated the *p* values of entropy analysis based on the null hypothesis of “entropy of actual pattern is larger than entropies of shuffled patterns.” We should mention that we carried out all statistical analyses in R software (Kassambara, 2020; Mangiafico & Mangiafico, 2017; RC Team, 2013; Whitcher et al., 2013). We provided Figure 1 using “draw.io” and statistical figures and brain maps by the advance of “BrainNet Viewer” (Xia et al., 2013) and some other R packages (Nakazawa, 2019; Wickham et al., 2016). In addition, we shared all of the information and codes at https://github.com/majidsaberi/BrainNetFrustration (Saberi, 2022) so everyone can publicly access, replicate, and develop our research.

**Table 2.** Effect size thresholds and corresponding algorithms

Nonparametric test	Effect size algorithm	R package	Small	Medium	Large
Paired two-group	Wilcoxonpairedr	R companian	0.1 – <0.4	0.4 – <0.6	≥0.6
Multiple-group	Kruskal_effsize	Rstatix	0.01 – <0.06	0.06 – <0.14	≥0.14

## ACKNOWLEDGMENTS

We would like to thank ABIDE and Southwest University for generously sharing the data and David Matthews for reading the manuscript and language checking.

## SUPPORTING INFORMATION

Supporting information for this article is available at https://doi.org/10.1162/netn_a_00268 and https://github.com/majidsaberi/BrainNetFrustration.

## AUTHOR CONTRIBUTIONS

Majid Saberi: Conceptualization; Formal analysis; Methodology; Project administration; Writing – original draft. Reza Khosrowabadi: Validation; Visualization; Writing – review & editing. Ali Khatibi: Validation; Visualization; Writing – review & editing. Bratislav Mišić: Validation; Visualization; Writing – review & editing. Gholamreza Jafari: Validation; Visualization; Writing – review & editing.

## Supplementary Material





## TECHNICAL TERMS

Signed network: A network where links have positive and negative attributes, e.g., a social network with friendship and hostility links.
Structural balance theory: A mathematical framework for studying the balance of relationships in a system of entities with positive and negative relations.
Social balance: When a friend’s friend or an enemy’s enemy is an enemy and everything is well.
Social imbalance: When a friend’s friend or an enemy’s enemy is an enemy. It is nontrivial; entities endure a tension and tend to change their relationships.
Signed links: Network links with positive and negative qualities.
Frustration: A topological constraint that prevents system energy minimization, it brings flexibility to the system.
Balance theory: An attitude change theory developed by Fritz Heider that explains how the quality of relationships affects people’s mind-sets.
Brain signed network: A brain network made based on functional neuroimage data where synchronous and antisynchronous brain coactivations determine the positive and negative links between brain nodes.
Network frustration: A closed cycle of signed links having an odd number of negative links and frustrating properties.

## Supporting Information

https://github.com/majidsaberi/BrainNetFrustration
